# Redescription and establishment of a holotype and three paratypes for the species *Hemimycale mediterranea* sp. nov.

**DOI:** 10.7717/peerj.3426

**Published:** 2017-06-08

**Authors:** Maria J. Uriz, Leire Garate, Gemma Agell

**Affiliations:** Department of Marine Ecology, Centre for Advanced Studies of Blanes (CEAB-CSIC), Blanes, Girona, Spain

**Keywords:** *Hemimycale mediterranea*, Holotype, Paratypes, Nomenclature

## Abstract

**Background:**

In a recent paper, we described a new sponge species named *Hemimycale mediterranea* Uriz, Garate & Agell, 2017. However, we failed to designate a holotype and a type locality, as required by the International Commission on Zoological Nomenclature (ICZN). Although the validity of the previous conclusions remains unchanged, the species name cannot be considered available according to ICZN regulations until a holotype is designated.

**Results:**

The present work fulfills the requirements of the ICZN by designating a holotype, three paratypes and the type locality for the new species *Hemimycale mediterranea* and has been registered in ZooBank.

## Introduction

In an earlier version of this article published on 7 March 2017, Uriz, Garate & Agell (2017) reassessed the taxonomy of the genus *Hemimycale* Burton, 1934 (Porifera: Poecilosclerida), and describe a new species of *Hemimycale,* which is morphologically cryptic with the Atlanto-Mediterranean *Hemimycale columella*. The new species, named *Hemimycale mediterranea,* was extensively described in the above mentioned paper by Uriz, Garate & Agell (2017) (LSID: urn:lsid:zoobank.org:pub:69255188-5A55-4D5C-9DC2-43E2B6CF6997) based on morphological and molecular characters, but the authors did not include a designation of the holotype specimen of *Hemimycale mediterranea*. Therefore, this nomenclatural act cannot be considered as published under International Commission on Zoological Nomenclature (ICZN) regulations, and the species name is not available from the earlier version of this work.

In the present work, we designate a holotype and three paratypes and indicate the type locality for the new species. Similarly, this work has been registered in ZooBank (see below) and now fulfills the requirements of the ICZN for a holotype designation.

## Material and Methods

The electronic version of this article in, Portable Document Format (PDF), will represent a published work according to the ICZN. Hence, the holotype and paratype designation contained in the electronic version is effectively published under the ICZN code from the electronic edition alone. This published work has been registered in ZooBank, the online registration system for the ICZN. The ZooBank LSID (Life Science Identifier) can be resolved and the associated information viewed through any standard web browser by appending the LSID to the prefix “http://zoobank.org/”. The LSID for this publication is: urn:lsid:zoobank.org:pub:E2F883E3-FDAD-4F2A-A82C-28AF03C55C8C.

The online version of this work is archived and available from the following digital repositories: PeerJ.

## Species Systematics

**Table:** 

Phylum Porifera Grant, 1836
Class Demospongiae Sollas, 1885
Order Poecilosclerida Topsent, 1928
Family Hymedesmiidae Topsent, 1928 (see Van Soest, 2002)
Genus *Hemimycale* Burton, 1934 (see Van Soest et al., 2016)
*Hemimycale mediterranea* sp. nov.

**Taxonomic assessment**

The name *Hemimycale mediterranea* published in an earlier version of this article on 7 March 2017, is not available from the earlier version of this work because it did not include a designation of the holotype.

**Type material**

CRBA-56057 is the sponge specimen herein designated as holotype (see Remarks, below). CRBA-56058-60 are three specimens here designated as paratypes. The holotype and the paratypes have been deposited at the Centre de Recursos de Biodiversitat Animal (Faculty de Biology), University of Barcelona, Spain.

**Type locality**

The locality from which the holotype was collected was “Roca del Moro” (41.7074°N; 2.91172°E) (Muladera), in Tossa de Mar, at 15 m of depth.

**Remarks**

A complete description of the new species *Hemimycale mediterranea* is provided by Uriz, Garate & Agell (2017), and we reference that publication in accordance with Article 13.1.2 of the ICZN Code. However, no holotype was designated for the new species in the previous publication. In order to avoid potential future issues with the taxonomic status of *H. mediterranea*, the specimens here described are herein designated as the species holotype (CRBA-56057) and paratypes (CRBA.56058, CRBA.56059, CRBA.56060), ZooBank LSID: urn:lsid:zoobank.org:pub:E2F883E3-FDAD-4F2A-A82C-28AF03C55C8C.

## Description

Species: *H. mediterranea* sp. nov.

GenBank accession Numbers of sequences (Table 1).

**Table 1:** Geographical origin and ecological distribution of the individuals of *Hemimycale mediterranea* analyzed with GenBank accession numbers. Holotype and paratypes are in bold.

Individuals	Sea/Ocean	Locality	Voucher numbers	Accession numbers
***H. mediterranea*sp. nov. ind. 1**	Northwestern Mediterranean	Tossa de Mar-Spain	**Holotype: CRBA-56057**	COI: KY002130 18S: KY002162 28S: KY002189
***H. mediterranea*sp. nov. ind. 2**	Northwestern Mediterranean	Tossa de Mar-Spain	**Paratype:CRBA-56057**	18S: KY002163 28S: KY002190
***H. mediterranea*sp. nov. ind. 4**	Northwestern Mediterranean	Tossa de Mar-Spain	CEAB.POR.GEN.012	COI: KY002131
***H. mediterranea*sp. nov. ind. 5**	Northwestern Mediterranean	Tossa de Mar-Spain	CEAB.POR.GEN.013	COI: KY002132
***H. mediterránea*sp. nov. ind. 3**	Adriatic Sea	Koznati-Croatia	CEAB.POR.GEN.014	COI: KY002134
***H. mediterránea*sp. nov. ind. 7**	Adriatic Sea	Koznati-Croatia	CEAB.POR.GEN.015	18S: KY002170 28S: KY002193
***H. mediterránea*sp. nov. ind. 8**	Adriatic Sea	Koznati-Croatia	CEAB.POR.GEN.016	28S: KY002194
***H. mediterránea*sp. nov. ind. 2**	Adriatic Sea	Tremity-Italy	**Paratype:CRBA-56060**	COI: KY002133
***H. mediterránea*sp. nov. ind. 11**	Adriatic Sea	Tremity-Italy	**Paratype:CRBA-56059**	28S: KY002199
***H. mediterránea*sp. nov. ind. 8**	Central Mediterranean	Porto Cesareo-Italy	CEAB.POR.GEN.019	18S: KY002164
***H. mediterránea*sp. nov. ind. 9**	Central Mediterranean	Porto Cesareo-Italy	CEAB.POR.GEN.020	18S: KY002165 28S: KY002197
***H. mediterránea*sp. nov. ind. 10**	Central Mediterranean	Porto Cesareo-Italy	CEAB.POR.GEN.021	28S: KY002198
***H. mediterránea*nov. sp**. **ind. 5**	Adriatic Sea	Karaburum-Albania	CEAB.POR.GEN.022	18S: KY002166 28S: KY002191
***H. mediterránea*nov. sp**. **ind. 6**	Adriatic Sea	Karaburum-Albania	CEAB.POR.GEN.023	18S: KY002167 28S: KY002192
***H. mediterránea*sp. nov. ind. 3**	Eastern Mediterranean	Othonoi-Greece	CEAB.POR.GEN.024	18S: KY002168 28S: KY002195
***H. mediterránea*sp. nov. ind. 4**	Eastern Mediterranean	Othonoi-Greece	CEAB.POR.GEN.025	18S: KY002169 28S: KY002196

**Figure 1:**
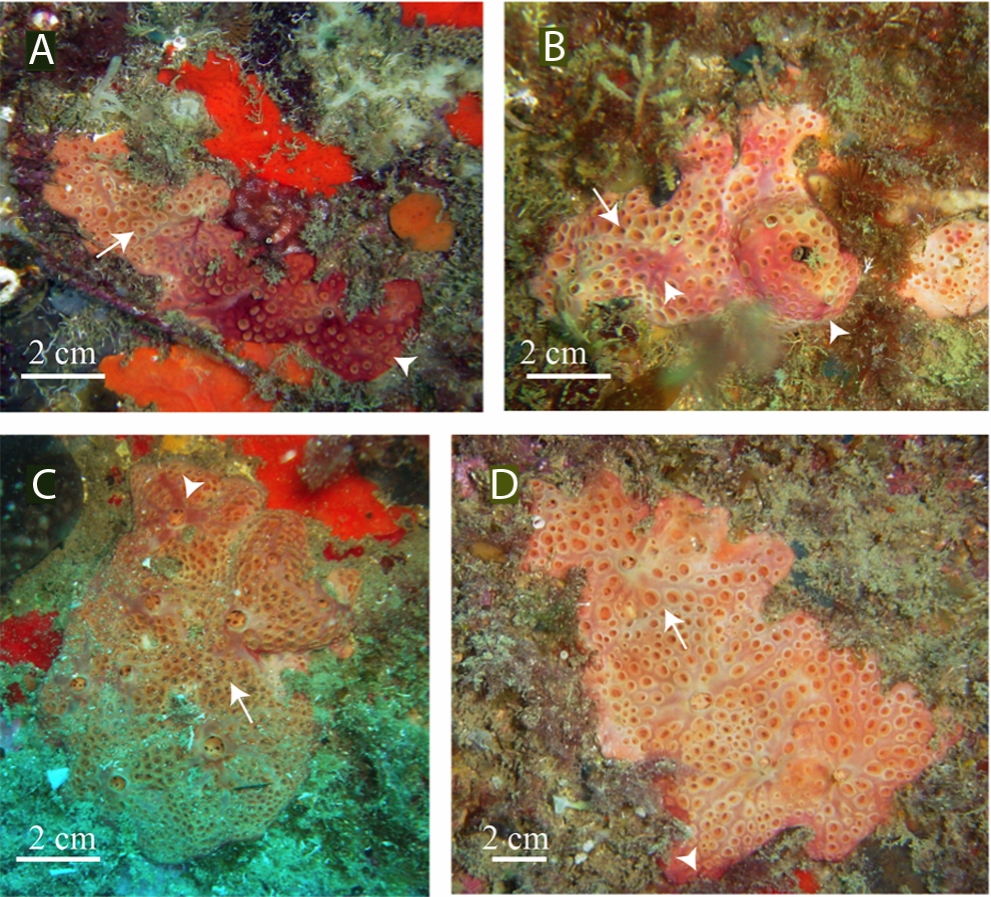
*In situ* pictures of *Hemimycale mediterranea* sp. nov. from 12–17 m of depth. Whitish tinge is due to calcibacteria accumulation. Red tinges are due to several species of epibiotic cyanobacteria. Arrows point to aerolate inhaling areas; arrowheads indicate the epibiont cyanophycea.

Description: thick encrusting sponges with aerolate inhaling areas up to 3 mm in diameter, surrounded by an up to 1.5–2 mm high rim that in some cases barely surpasses the sponge surface (Fig. 1). Thousands of calcareous spherules, 1 µm in diameter, formed by intracellular calcifying bacteria, are spread through the sponge mesohyl and especially accumulated at the sponge periphery (Uriz et al., 2012; Garate et al., 2017).

Ectosome: firmly attached to the choanosome.

Color: flesh to clear brownish externally, with whitish tinges depending on calcibacteria accumulation at the surface that was sometimes partially covered by an epibiotic (reddish or pinkish) cyanobacteria.

**Figure 2:**
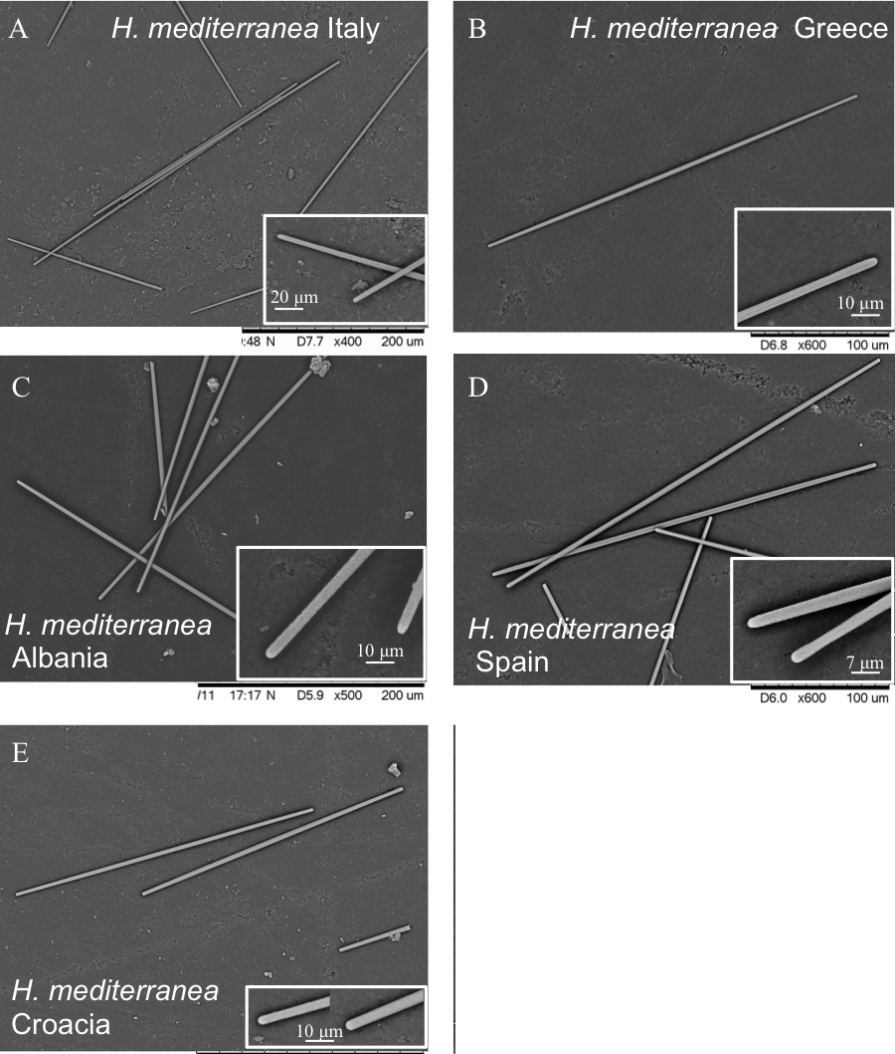
Spicules (anisostrongyles) of various individuals of *Hemimycale mediterranea* sp. nov. though SEM from several localities.

Spicules (Table 2, Fig. 2): smooth, uniform in size, straight, anysostrongyles, 200–296 × 3–4 µm in size. Styles completely absent.

Skeletal arrangement: plumose undulating bundles of anysostrongyles together with spread spicules. A palisade of vertical anysotrongyles forms the rim around the inhaling areas.

Known distribution: northwestern Mediterranean, central Mediterranean, Adriatic, eastern Mediterranean (Spain: Cap De Creus, Tossa, Blanes, Arenys, South Italy: Croatia, Tremiti, Turkey, Greece), between 3 and 17 m deep.

**Table 2:** Localities and spicule sizes of the studied individuals of *Hemimycale mediterranea*.

Species	Author	Locality	Depth (m)/ Assemblage	Styles	Strongyles (range/mean)
*H. mediterránea* ind. 7	Uriz, Garate & Agell (2017)	Adriatic (Croatia)	10–15/rocky sub-horizontal	_	233–330 (274.8) × 3–4.6 (4.0)
*H. mediterránea* ind. 11	Uriz, Garate & Agell (2017)	Adriatic (Italy)	10–15/rocky sub-horizontal	_	251–300 (276.6) × 2.1–4 (3.0)
*H. mediterránea* ind. 5	Uriz, Garate & Agell (2017)	Adriatic (Albania)	10–15/rocky sub-horizontal	_	274–317 (296.4) × 2.9–4.5 (4.0)-
*H. mediterránea* ind. 10	Uriz, Garate & Agell (2017)	Central Med. (Italy)	10–15/rocky sub-horizontal	_	229–328 (291.3) × 2.4–5.2 (3.5)
*H. mediterránea* ind. 3	Uriz, Garate & Agell (2017)	Eastern Med. (Greece)	10–15/rocky sub-horizontal	_	242–340 (272.7) × 2.6–4 (3.2)
*H. mediterránea* ind. 1	Uriz, Garate & Agell (2017)	NW Med. (Spain)	12–16/rocky wall	_	261–320(296.3) × 3.1–3.8 (3.5)

Biology: the species has an annual life span with maximum growth rates in summer (M Uriz, L Garate & G Agell, 2012, unpublished data). Larval release occurs at the end of September and beginning of October (Pérez-Porro, González & Uriz, 2012).

## Additional Information and Declarations

### DNA Deposition

The following information was supplied regarding the deposition of DNA sequences: GenBank: COI: KY002130
18S: KY002162
28S: KY002189
18S: KY002163
28S: KY002190
COI: KY002131
COI: KY002132
COI: KY002134
18S: KY002170
28S: KY002193
28S: KY002194
COI: KY002133
28S: KY002199
18S: KY002164
18S: KY002165
28S: KY002197
28S: KY002198
18S: KY002166
28S: KY002191
18S: KY002167
28S: KY002192
18S: KY002168
28S: KY002195
18S: KY002169
28S: KY002196.

### Data Availability

The following information was supplied regarding data availability: GenBank: 18S, 28S and COI sequences are accessible at https://www.ncbi.nlm.nih.gov/genbank/.

### New Species Registration

The following information was supplied regarding the registration of a newly described species: Species name: *Hemimycale mediterranea* Publication LSID: urn:lsid:zoobank.org:pub:E2F883E3-FDAD-4F2A-A82C-28AF03C55C8C.
